# Supplementary material for: Neurocomputational mechanisms of confidence in self and others
Source: Nat Commun. 2022 Jul 22;13:4238. doi: 10.1038/s41467-022-31674-w (PMC9307648; doi:10.1038/s41467-022-31674-w)
Supplement: Supplementary file 1 — Supplementary Information [file 41467_2022_31674_MOESM1_ESM.pdf]

Supplementary information

Neurocomputational mechanisms of confidence in self and others

Dan Bang*, Rani Moran, Nathaniel D. Daw & Stephen M. Fleming*

*Correspondence: danbang.db@gmail.com and stephen.fleming@ucl.ac.uk

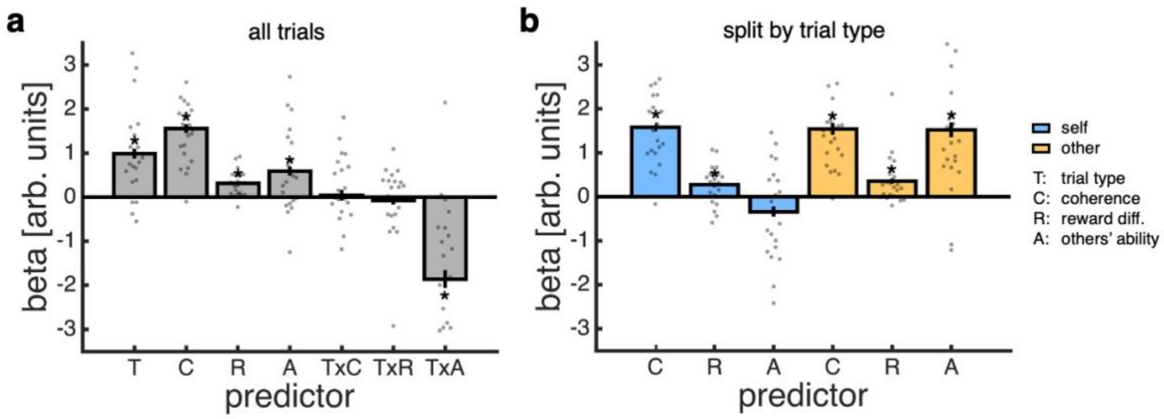

Supplementary Fig. 1. Multiple logistic regression analysis of post-decision wagers. **a** We first predicted gamble choices (i.e., selecting the risky option over the safe option) on all trials using trial type (T; other = -.5; self = .5), coherence (C; z-score), reward difference (R; z-score), others' ability (A; low = -.5, medium = 0, high = .5) and the interactions between trial type and the three other terms (TxC, TxR and TxA). The results were as follows: T, $t(20) = 4.47$, $p < .001$; C, $t(20) = 7.78$, $p < .001$; R, $t(20) = 5.02$, $p < .001$; A, $t(20) = 2.85$, $p = .010$; TxC, $t(20) = 0.18$, $p = .858$; TxR, $t(20) = -0.44$, $p = .664$; TxA, $t(20) = -4.60$, $p < .001$. **b** We then predicted gamble choices separately for self-trials (blue) and other-trials (yellow) using coherence, reward difference and others' ability. The results for self-trials were as follows: C, $t(20) = 8.17$, $p < .001$; R, $t(20) = 2.88$, $p = .009$; A, $t(20) = -1.46$, $p = .161$. The results for other-trials were as follows: C, $t(20) = 5.74$, $p < .001$; R, $t(20) = 2.91$, $p = .009$; A, $t(20) = 4.52$, $p < .001$. **a-b** We performed separate regressions for each participant. We tested group-level significance (asterisk) by comparing coefficients pooled across participants to zero (one-sample *t*-test). Data are represented as group mean ± SEM, $n = 21$. Individual participants are overlaid as dots. Source data are provided as a Source Data file.

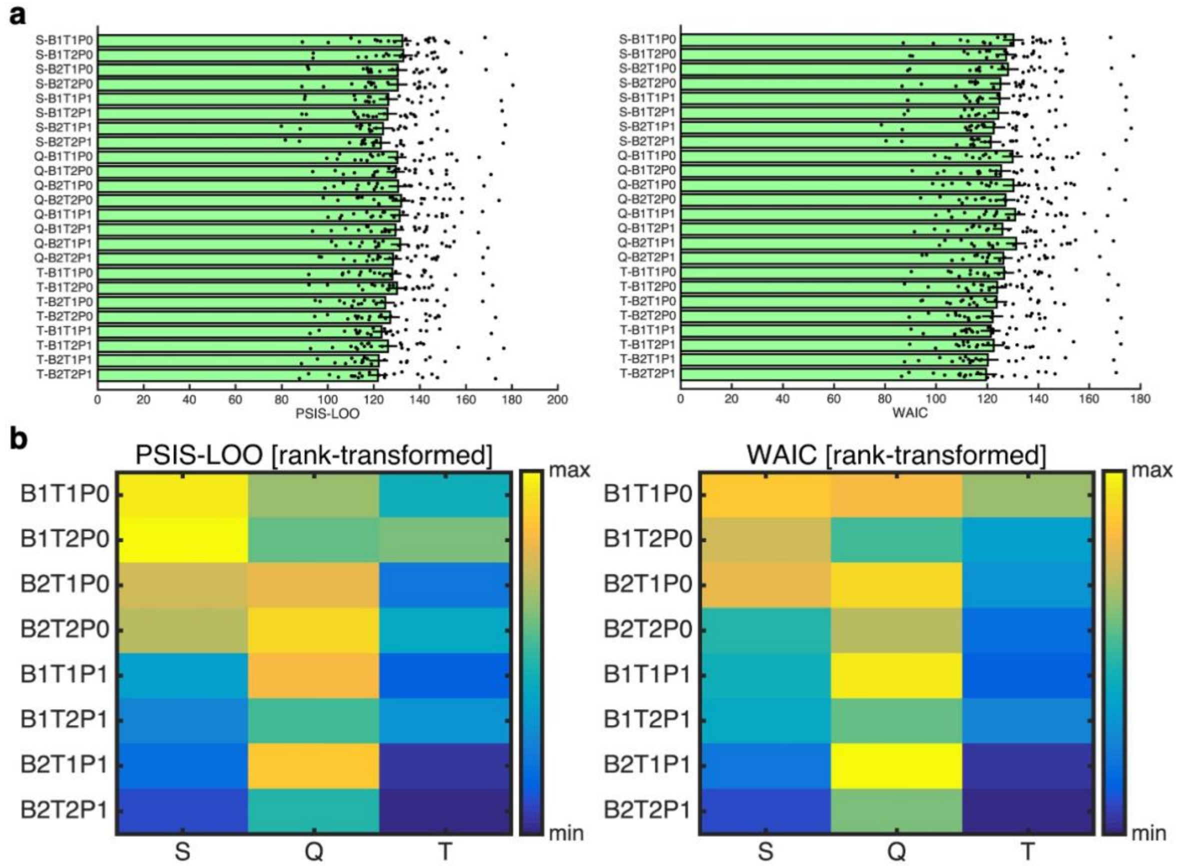

Supplementary Fig. 2. Model comparison. The model comparison was based on leave-one-out cross-validation using (left) Pareto-smoothed importance sampling (PSIS-LOO) or (right) the widely applicable information criterion (WAIC). In both cases, smaller values indicate better fit. **a** Metrics for each model represented as group mean ± SEM, n = 21. Individual participants are overlaid as dots. **b** Rank-transformed average model metrics. **a-b** Model classes are abbreviated as follows: S, S-model; Q, Q-model; T, ToM-model. Model versions are abbreviated as follows: B, softmax bias (1: same bias for self and other; 2: different bias for self and other); T, softmax temperature (1: same temperature for self and other; 2: different temperature for self and other); P, rescaling of stimulus space (0: linear spacing; 1: re-scaled according to fitted factor). See Supplementary Table 1 for overview of model parameters. Source data are provided as a Source Data file.

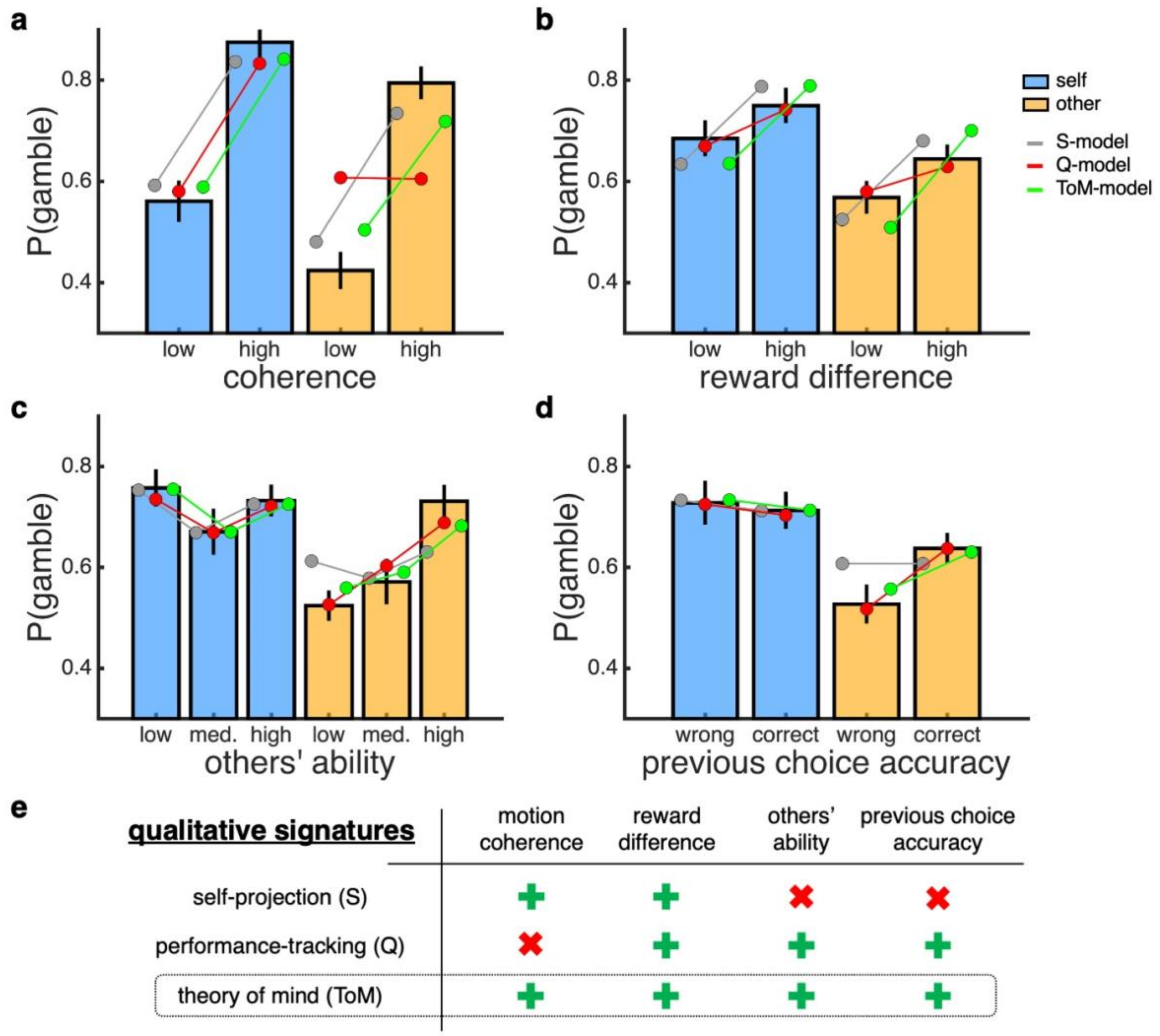

Supplementary Fig. 3. Model predictions. In each panel, data are split into self-trials (blue) and other-trials (yellow). **a** Probability gamble (i.e., proportion of trials in which the risky option was selected) as a function of coherence (median split). **b** Probability gamble as a function of the reward difference between the risky and the safe option (median split). **c** Probability gamble as a function of the ability of the other player. **d** Probability gamble as a function of choice accuracy on the previous trial of the same type. **e** While all models predict the observed pattern of results on self-trials, only the ToM-model predicts the observed pattern of results on other-trials. **a-d** Bar charts are empirical data. Dots show data simulated under the best-fitting version of each model class (S-B2T2P1, Q-B2T2P1, ToM-B2T2P1). Models are denoted by colour and displayed in the following order: S-model (grey), Q-model (red) and ToM-model (green). Empirical data are represented as group mean ± 95% CI and model predictions are represented as group mean, n = 21.

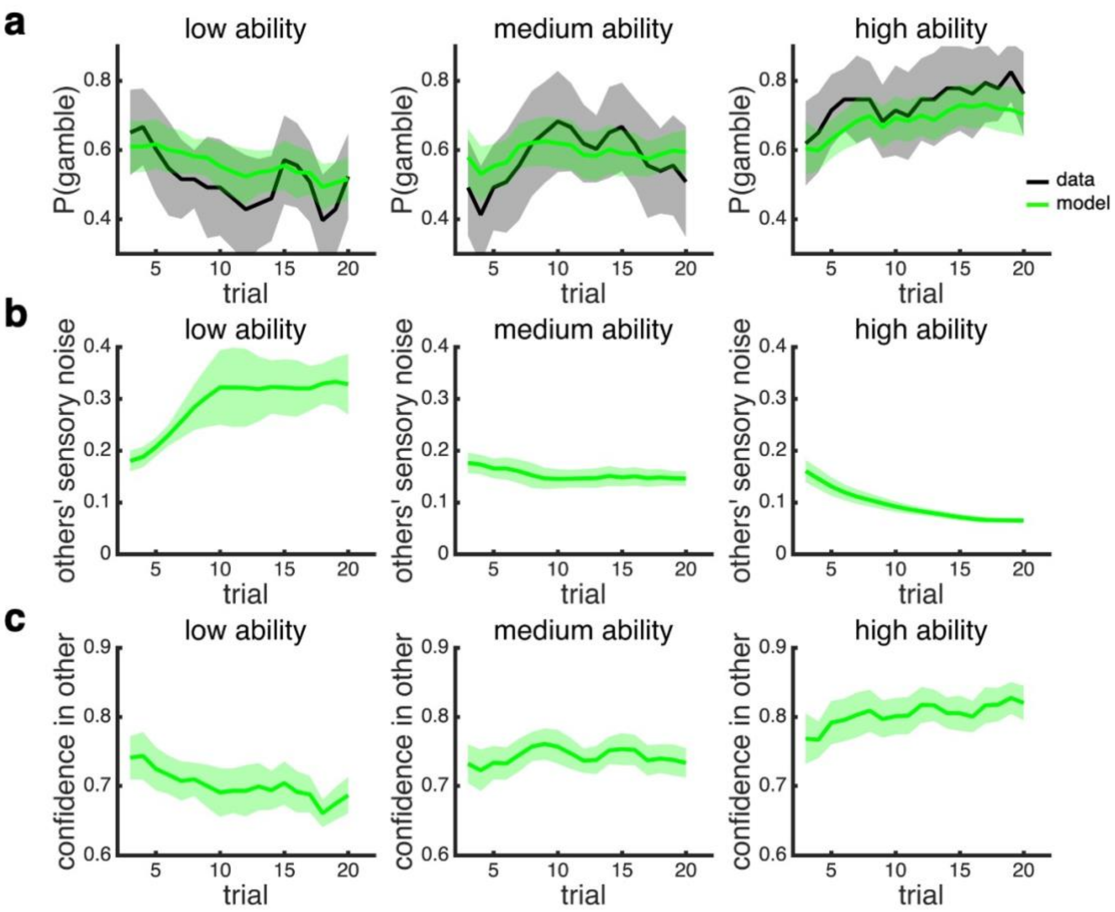

Supplementary Fig. 4. Temporal dynamics of model variables for the three other players. **a** Evolution of probability gamble (i.e., proportion of trials in which the risky option was selected) as observed in the behavioural data and as predicted under the ToM-model. **b** Evolution of the estimate of the other players' sensory noise under the ToM-model. **c** Evolution of confidence in the other players' choice under the ToM-model. For each variable, we applied a running average where the value on trial *t* was defined as the average over trials *t*-2 to *t*; the data are therefore shown from trial 3 onwards. Empirical and simulated data are represented as group mean ± 95% CI, *n* = 21.

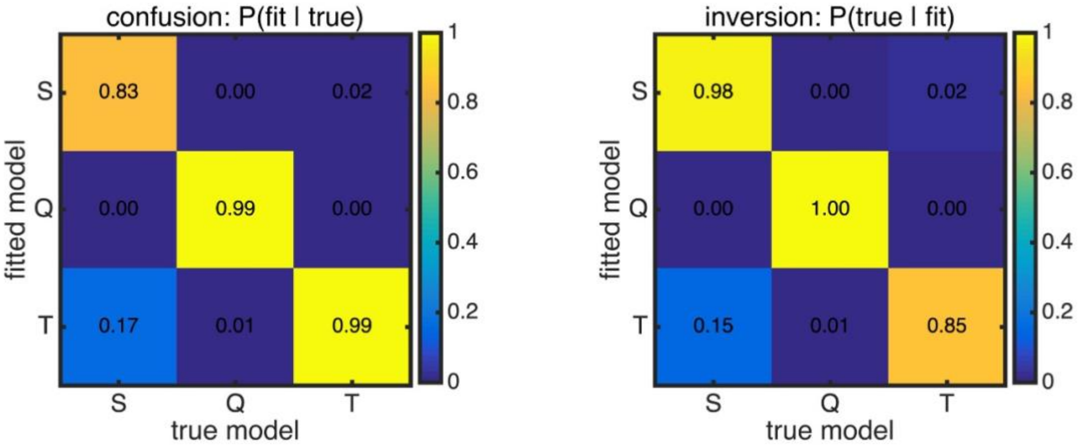

Supplementary Fig. 5. Model recoverability analysis. We selected the best-fitting version of each model class (S-B2T2P1, Q-B2T2P1, ToM-B2T2P1), simulated 200 datasets for each model and then fitted the models to the simulated datasets; each dataset mirrored our study in terms of participants, task variables and trials. Based on the model comparison results (WAIC) for all 200 iterations, we computed a confusion and an inversion matrix. A confusion matrix shows the probability that model Y provided the best fit to data generated by model X. By contrast, an inversion matrix shows the probability that model X generated the data given that model Y provided the best fit. We calculated the inversion matrix directly from the confusion matrix and assumed that the three models were a priori equally likely. Model classes are abbreviated as follows: S, S-model; Q, Q-model; T, ToM-model.

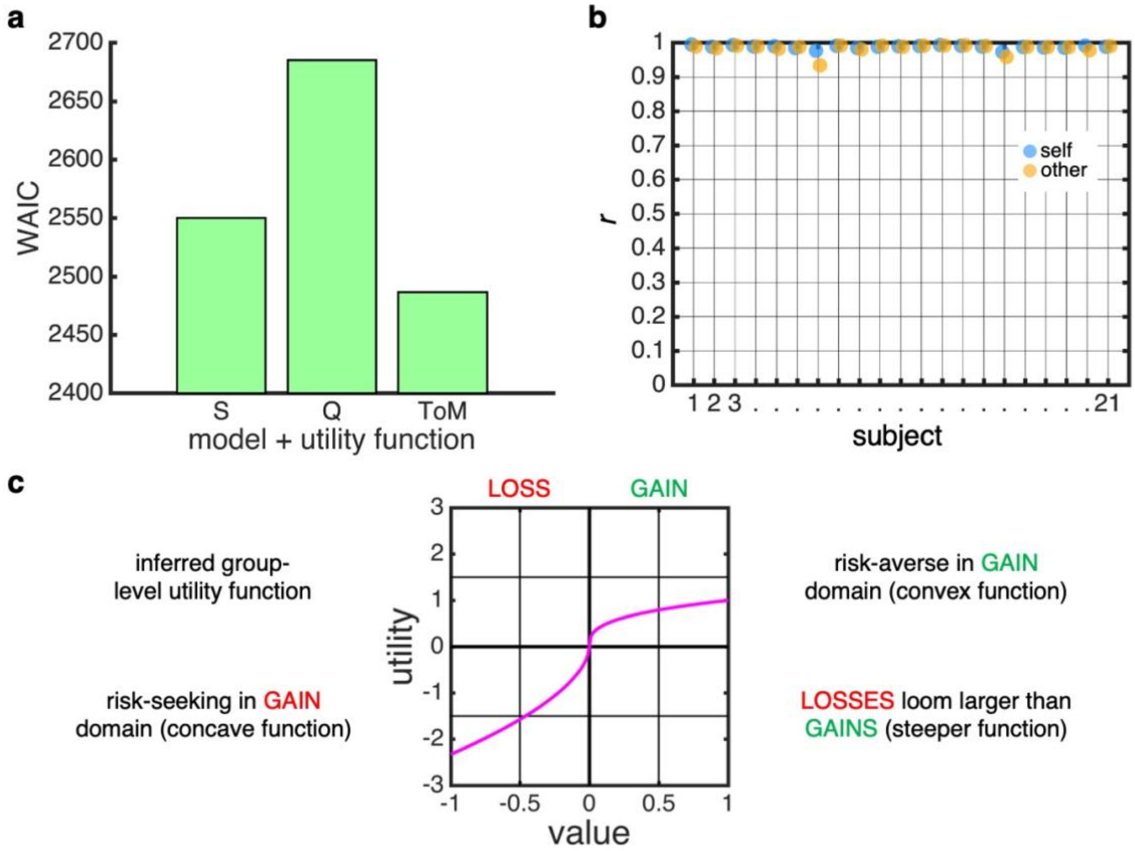

Supplementary Fig. 6. Risk and/or loss aversion. To assess the potential impact of risk and/or loss aversion on the model-based confidence estimates, we ran a control analysis in which we re-fitted the best-fitting version of each model class (S-B2T2P1, Q-B2T2P1, ToM-B2T2P1) after including a utility function. **a** The ToM-model still provided the best account of the behavioural data (sum of WAIC across participants) – indicating that value-based processes did not bias our model comparison results. **b** The model-based confidence estimates under both the original ToM-model and the ToM-model with a utility function were highly correlated (Pearson's *r*) within every participant – supporting that the original ToM-model was sufficient to infer participants' confidence from their PDWs. **c** Visualisation of the inferred group-level utility function obtained from the fit of the augmented ToM-model. We normalised the value range for ease of visualisation. Source data are provided as a Source Data file.

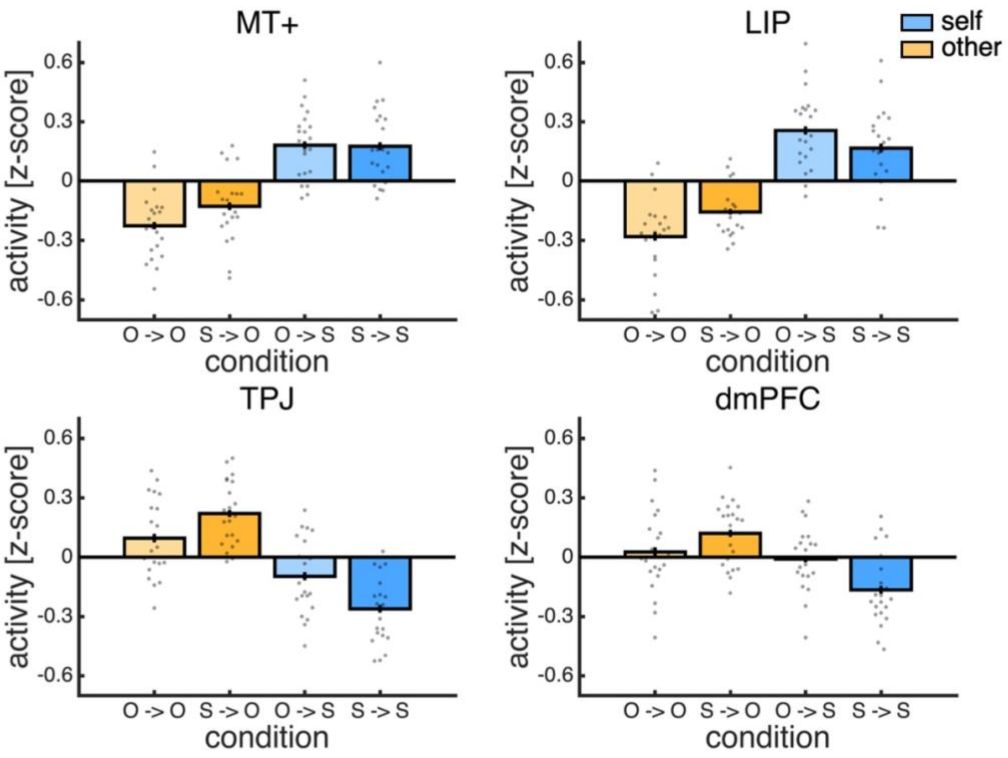

Supplementary Fig. 7. Repetition suppression analysis of agent-specific effects in sensory and social ROIs. In each panel, the z-scored stimulus-related activity estimates under the canonical hemodynamic response function are split according to the current trial type (yellow: other, O; blue: self, S) and whether the previous trial type was the same (light: same; dark: different). In MT+, there was only an effect of the current trial type (linear regression: current, $t(20) = 7.93$, $p < .001$; previous, $t(20) = 1.18$, $p = .252$; interaction, $t(20) = -1.10$, $p = .284$). However, in addition to an effect of the current trial type, there was an interaction between the previous and the current trial type in LIP (linear regression: current, $t(20) = 7.73$, $p < .001$; previous, $t(20) = 0.36$, $p = .719$; interaction, $t(20) = -3.42$, $p = .003$), TPJ (linear regression: current, $t(20) = -6.16$, $p < .001$; previous, $t(20) = -0.52$, $p = .609$; interaction, $t(20) = -3.40$, $p = .003$) and dmPFC (linear regression: current, $t(20) = -3.61$, $p = .002$; previous, $t(20) = -0.66$, $p = .516$; interaction, $t(20) = -2.80$, $p = .011$). Under the repetition suppression framework, if a region is primarily involved in self-inference, then activity should be highest on self-trials preceded by other-trials, and lowest on other-trials preceded by other-trials – a pattern observed for LIP. By contrast, if a region is primarily involved in other-inference, then activity should be highest on other-trials preceded by self-trials, and lowest on self-trials preceded by self-trials – a pattern observed for TPJ and dmPFC. Data are represented as group mean ± SEM, $n = 21$. Individual participants are overlaid as dots. Source data are provided as a Source Data file.

parameter	range	sampling	note
**sensation (all)**			
noise, σ	[.01,2]	$\sigma \sim normal(a, b)$ where a and b are obtained from a posterior distribution over sensory noise as fitted in the prescan session	
sensory sample, x	[-1,1]	$x \sim normal(a, b)$ where a is the motion stimulus, $k\theta$, and b is noise, σ	
**softmax (all)**			
bias, β_0	[-10,10]	$\beta_0 \sim normal(a, b)$, where a and b are group-level parameters, $a \sim normal(0,10)$ and a: $[-10,10]$, $b \sim uniform(.01,10)$	same (B1) or different (B2) bias for self and other
temperature, β_1	[.01,2]	$\beta_1 \sim normal(a, b)$, where a and b are group-level parameters, $a \sim normal(.2,2)$ and a: $[.01,2]$, $b \sim uniform(.01,2)$	same (T1) or different (T2) temperature for self and other
**stimulus space**			
factor, p	[.01,10]	$p \sim normal(a, b)$, where a and b are group-level parameters, $a \sim normal(2,4)$ and a: $[.01,10]$, $b \sim uniform(.01,4)$	linearly-spaced (P0) or re-scaled (P1) stimulus space
**learning (Q, T)**			
alpha, α	[0,1]	$\alpha \sim beta(a, b)$, where a and b are group-level parameters, $a = x * y^{-2}$, $x \sim normal(.2,1)$ and x: $[0,1]$, $y \sim uniform(0,2)$, $b = y^{-2} - a$	
**Utility (S, Q, T)**			
risk loss, R		$R \sim normal(a, b)$, where a and b are group-level parameters, $a \sim normal(.6,1)$ and a: $[.01,1]$, $b \sim uniform(.01,1)$	
risk gain, T		$T \sim normal(a, b)$, where a and b are group-level parameters, $a \sim normal(.4,1)$ and a: $[.01,1]$, $b \sim uniform(.01,1)$	
loss aversion, S		$S \sim normal(a, b)$, where a and b are group-level parameters, $a \sim normal(1,4)$ and a: $[.01,10]$, $b \sim uniform(.01,10)$	

Supplementary Table 1. Overview of model parameters and priors as implemented in STAN. Model classes are abbreviated as follows: S, S-model; Q, Q-model; T, ToM-model. See online repository for code implementation. Here '∼' indicates 'is distributed as' and ':' indicates 'range'.

contrast	label	voxels at $p < .001$	peak z-score	p (cluster, FWE-corrected)	peak voxel MNI coordinates			
self > other	extrastriate, parietal, motor, cingulate	23632	6.25	<.001	-34	-16	68	LR
	thalamus	252	5.95	<.001	-22	-26	-6	L
	thalamus	145	5.49	<.001	22	-26	4	R
	inferior frontal gyrus	116	5.22	.002	-58	6	26	L
	insula	656	5.04	<.001	42	0	8	R
	striatum	222	4.40	<.001	12	18	-8	R
	striatum	192	4.36	<.001	18	-10	22	R
other > self	dorsomedial frontal	1941	5.30	<.001	14	30	52	LR
	temporoparietal	892	5.30	<.001	-48	-56	26	L
	temporoparietal	741	5.23	<.001	50	-62	36	R
	temporal	848	4.91	<.001	-58	-38	0	L
	precuneus	607	4.85	<.001	2	-60	38	LR
	middle frontal gyrus	177	4.75	<.001	48	24	36	R
	temporal	525	4.42	<.001	66	-28	-8	R
	middle frontal gyrus	467	4.41	<.001	-36	6	46	L
	temporal	82	3.85	.014	58	-8	-30	R

Supplementary Table 2. Summary of significant activations for GLM1: decision phase. Group-level significance was assessed by applying one-sample t-tests against zero to the first-level contrast images as implemented by SPM. All reported activations are significant at $p < .05$, FWE-corrected for multiple comparisons. Cluster-defining threshold: $p < .001$, uncorrected. FWE: familywise error. MNI: Montreal Neurological Institute. L: left. R: right.

contrast	label	voxels at $p < .001$	peak z-score	p (cluster, FWE-corrected)	peak voxel MNI coordinates			
self > other	posterior cingulate	87	4.16	.009	-12	-52	10	LR
	ventromedial prefrontal	66	3.95	.037	2	38	-4	LR
other > self	middle frontal gyrus	258	5.44	<.001	-42	10	48	L
	temporoparietal	719	5.30	<.001	-48	-58	24	L
	temporal	503	5.30	<.001	64	-24	-12	R
	temporoparietal	550	5.23	<.001	54	-60	30	R
	dorsomedial frontal	1751	5.04	<.001	0	32	52	LR
	temporal	467	5.01	<.001	-64	-46	-2	L
	precuneus	176	4.96	<.001	6	-66	42	LR
	middle frontal gyrus	316	4.74	<.001	44	22	38	R
	temporal	414	4.68	<.001	-50	12	-28	L
	orbitofrontal	140	4.63	<.001	42	52	-12	R
	orbitofrontal	529	4.48	<.001	-34	54	-10	L
	orbitofrontal	97	4.44	.004	52	22	-6	R
	cerebellum	107	4.43	.002	-8	-80	-28	LR
	orbitofrontal	177	4.36	<.001	-28	22	-8	L
	precuneus	83	4.26	.011	6	-52	34	LR
	orbitofrontal	89	4.21	.007	-10	48	-24	LR
	temporal	84	4.17	.010	58	-38	2	R
	cerebellum	116	4.16	.001	-30	-84	-30	L
	orbitofrontal	75	3.98	.019	30	18	-6	R

Supplementary Table 3. Summary of significant activations for GLM1: gamble phase. Group-level significance was assessed by applying one-sample t-tests against zero to the first-level contrast images as implemented by SPM. All reported activations are significant at $p < .05$, FWE-corrected for multiple comparisons. Cluster-defining threshold: $p < .001$, uncorrected. FWE: familywise error. MNI: Montreal Neurological Institute. L: left. R: right.
