# Supplementary material for: Neurocomputational mechanisms of confidence in self and others
Source: Nat Commun. 2022 Jul 22;13:4238. doi: 10.1038/s41467-022-31674-w (PMC9307648; doi:10.1038/s41467-022-31674-w)
Supplement: Supplementary file 3 — Reporting Summary [file 41467_2022_31674_MOESM3_ESM.pdf]

Corresponding author(s): Dan Bang, Stephen M. Fleming

Last updated by author(s): Apr 26, 2022

Reporting Summary

Nature Research wishes to improve the reproducibility of the work that we publish. This form provides structure for consistency and transparency in reporting. For further information on Nature Research policies, see our Editorial Policies and the Editorial Policy Checklist.

Statistics

For all statistical analyses, confirm that the following items are present in the figure legend, table legend, main text, or Methods section.

n/a	Confirmed	
☐	☒	The exact sample size (*n*) for each experimental group/condition, given as a discrete number and unit of measurement
☐	☒	A statement on whether measurements were taken from distinct samples or whether the same sample was measured repeatedly
☐	☒	The statistical test(s) used AND whether they are one- or two-sided<br>*Only common tests should be described solely by name; describe more complex techniques in the Methods section.*
☐	☒	A description of all covariates tested
☐	☒	A description of any assumptions or corrections, such as tests of normality and adjustment for multiple comparisons
☐	☒	A full description of the statistical parameters including central tendency (e.g. means) or other basic estimates (e.g. regression coefficient) AND variation (e.g. standard deviation) or associated estimates of uncertainty (e.g. confidence intervals)
☐	☒	For null hypothesis testing, the test statistic (e.g. *F*, *t*, *r*) with confidence intervals, effect sizes, degrees of freedom and *P* value noted<br>*Give P values as exact values whenever suitable.*
☐	☒	For Bayesian analysis, information on the choice of priors and Markov chain Monte Carlo settings
☐	☒	For hierarchical and complex designs, identification of the appropriate level for tests and full reporting of outcomes
☐	☒	Estimates of effect sizes (e.g. Cohen's *d*, Pearson's *r*), indicating how they were calculated

Our web collection on statistics for biologists contains articles on many of the points above.

Software and code

Policy information about availability of computer code

Data collection	The experiment was programmed in MATLAB 2014b using Psychtoolbox 3.0.12. The behavioural data were recorded using MATLAB 2014b. The fMRI data were recorded using a 3T Siemens Allegra scanner.
Data analysis	Behavioural data were analysed using MATLAB 2015b (custom code). Neural data were analysed using MATLAB 2015b (custom code, SPM12 and the Generalised PPI toolbox 13.1). Computational models were fitted using RStudio 1.0.153 and Stan 2.19.1 (custom code).

For manuscripts utilizing custom algorithms or software that are central to the research but not yet described in published literature, software must be made available to editors and reviewers. We strongly encourage code deposition in a community repository (e.g. GitHub). See the Nature Research guidelines for submitting code & software for further information.

Data

Policy information about availability of data

All manuscripts must include a data availability statement. This statement should provide the following information, where applicable:

- Accession codes, unique identifiers, or web links for publicly available datasets
- A list of figures that have associated raw data
- A description of any restrictions on data availability

Behavioural and neural data supporting main analyses including all figures are freely available on GitHub: https://github.com/danbang/article-self-other. Unthresholded group-level statistical maps are available on NeuroVault: https://neurovault.org/collections/9553/.

Field-specific reporting

Please select the one below that is the best fit for your research. If you are not sure, read the appropriate sections before making your selection.

☐ Life sciences ☒ Behavioural & social sciences ☐ Ecological, evolutionary & environmental sciences

For a reference copy of the document with all sections, see nature.com/documents/nr-reporting-summary-flat.pdf

Behavioural & social sciences study design

All studies must disclose on these points even when the disclosure is negative.

Study description	This study investigated the neurocomputational basis of confidence in self and others. Quantitative data were acquired, including behavioural variables and fMRI measures.
Research sample	We analysed data from 21 participants (12 female, mean ± SD age = 22.6 ± 4.4 years) from the subject pool of New York University. The sample is not necessarily representative of the general population. The sample was a convenience sample.
Sampling strategy	A convenience sample was recruited through the subject pool of New York University. The sample size was based on comparable published fMRI studies of perceptual decision-making (e.g., Fleming et al., 2012, Journal of Neuroscience).
Data collection	The experiment was delivered using computer software. All behavioural data were recorded by a computer. All fMRI data were recorded by computer. During the experiments, the subjects were alone in the testing/scanner room, with the researcher present in an adjacent room. The study was a within-subject design and blinding of the researcher was therefore not necessary.
Timing	Data were acquired between June 2014 and December 2014.
Data exclusions	One subject was excluded due to poor performance in the pre-scan session (not included in the Research sample section).
Non-participation	No subjects dropped out or declined participation.
Randomization	The study focused on within-subjects effects and no randomisation into groups was required.

Reporting for specific materials, systems and methods

We require information from authors about some types of materials, experimental systems and methods used in many studies. Here, indicate whether each material, system or method listed is relevant to your study. If you are not sure if a list item applies to your research, read the appropriate section before selecting a response.

Materials & experimental systems

n/a	Involved in the study	
☒	☐	Antibodies
☒	☐	Eukaryotic cell lines
☒	☐	Palaeontology and archaeology
☒	☐	Animals and other organisms
☐	☒	Human research participants
☒	☐	Clinical data
☒	☐	Dual use research of concern

Methods

n/a	Involved in the study	
☒	☐	ChIP-seq
☒	☐	Flow cytometry
☐	☒	MRI-based neuroimaging

Human research participants

Policy information about studies involving human research participants

Population characteristics	See above.
Recruitment	Subjects were recruited through advertisements to the subject pool at New York University. Subjects provided written informed consent before taking part in the study.
Ethics oversight	The study was approved by the New York University's University Committee on Activities Involving Human Subjects.

Note that full information on the approval of the study protocol must also be provided in the manuscript.

Magnetic resonance imaging

Experimental design

Design type	Event-related, randomised trial sequence.
Design specifications	Each subject completed 3 scan runs with 40 trials in each scan run. Each trial lasted approximately 11 seconds. There was a 0-3 second jitter between the decision and the gamble phase of a trial.
Behavioral performance measures	On self-trials, we recorded (1) a binary perceptual choice, (2) choice reaction time and (3) a post-decision wager. On other-trials, we recorded (1) a post-decision wager.

Acquisition

Imaging type(s)	Functional and structural.
Field strength	3T.
Sequence & imaging parameters	T1-weighted structural images were acquired using a 3D MPRAGE sequence: 1 x 1 x 1 mm resolution voxels; 176 sagittal slices. BOLD T2*-weighted functional images were acquired using a Siemens epi2d BOLD sequence: 3 x 3 x 3 mm resolution voxels; 42 transverse slices, 64 x 64 matrix; TR = 2.24s; TE = 30ms; slice tilt = -30 degrees T > C; slice thickness = 3 mm; interleaved slice acquisition). Local field maps were recorded for distortion correction of the acquired EPI data.
Area of acquisition	Whole-brain.

Diffusion MRI ☐ Used ☒ Not used

Preprocessing

Preprocessing software	SPM12
Normalization	Each subject's structural image was segmented into grey matter, white matter and cerebral spinal fluid using a nonlinear deformation field to map it onto a template tissue probability map. These deformations were applied to both structural and functional images to create new images spatially normalised to the Montreal Neurological Institute (MNI) space and interpolated to 2 x 2 x 2 mm voxels. Normalized images were spatially smoothed using a Gaussian kernel with full-width half-maximum of 8 mm.
Normalization template	SPM12 MNI template.
Noise and artifact removal	Motion correction parameters estimated from the re-alignment procedure and their first temporal derivatives (12 regressors in total) were included as confounds in the first-level analysis for each subject.
Volume censoring	N/A.

Statistical modeling & inference

Model type and settings	Mass univariate: fixed-effects within subjects for first-level analysis and random-effects across subjects for second-level analyses.
Effect(s) tested	Mass univariate: Group-level effects were tested with one-sample t-tests using SPM12. The statistical inference was conducted using Gaussian random field theory as implemented in SPM12 to obtain clusters satisfying P<0.05, family-wise error (FWE) corrected at a cluster-defining threshold of P<0.001, uncorrected.

Specify type of analysis: ☐ Whole brain ☐ ROI-based ☒ Both

Anatomical location(s)	ROI masks for MT+ were created using a localiser scan: we created a group mask using a second-level contrast between dynamic and static motion, and then, for each subject, created a MT+ mask (8-mm sphere) around their peak activity inside the group mask. ROI masks for LIP, TPJ and dmPFC were created using published connectivity-based parcellation atlases: LIP was defined as the union of areas SPLD and SPLE in the atlas developed by Mars et al. (2012, Cerebral Cortex); TPJ was defined as area TPJp in the atlas developed by Mars et al. (2012, Cerebral Cortex); and dmPFC was defined as area 9 in the atlas developed by Neubert et al. (2015, PNAS). All ROI masks were bilateral.

Statistic type for inference (See Eklund et al. 2016)	Mass univariate: voxel-wise inference.
Correction	Mass univariate: clusters satisfying P<0.05, family-wise error (FWE) corrected at a cluster-defining threshold of P<0.001, uncorrected.

Models & analysis

n/a	Involved in the study
[ ]	[x] Functional and/or effective connectivity
[x]	[ ] Graph analysis
[x]	[ ] Multivariate modeling or predictive analysis

Functional and/or effective connectivity

We conducted a psychophysiological interaction analysis using the Generalised PPI toolbox 13.1 for SPM (http://www.nitrc.org/projects/gppi/).
